# Neurocognitive Impairments Are More Severe in the Binge-Eating/Purging Anorexia Nervosa Subtype Than in the Restricting Subtype

**DOI:** 10.3389/fpsyt.2018.00138

**Published:** 2018-04-16

**Authors:** Hiroko Tamiya, Atushi Ouchi, Runshu Chen, Shiho Miyazawa, Yoritaka Akimoto, Yasuhiro Kaneda, Ichiro Sora

**Affiliations:** ^1^Department of Psychiatry, Kobe University Graduate School of Medicine, Kobe, Japan; ^2^Department of Biological Psychiatry, Tohoku University Graduate School of Medicine, Sendai, Japan; ^3^Department of Information & Management Systems Engineering, Nagaoka University of Technology, Nagaoka, Japan; ^4^Department of Psychiatry, Iwaki Clinic, Tokushima, Japan

**Keywords:** anorexia nervosa restricting subtype, anorexia nervosa binge-eating/purging subtype, MCCB Japanese version, neurocognitive impairment, subtype personality characteristics

## Abstract

**Objective:** To evaluate cognitive function impairment in patients with anorexia nervosa (AN) of either the restricting (ANR) or binge-eating/purging (ANBP) subtype.

**Method:** We administered the Japanese version of the MATRICS Consensus Cognitive Battery to 22 patients with ANR, 18 patients with ANBP, and 69 healthy control subjects. Our participants were selected from among the patients at the Kobe University Hospital and community residents.

**Results:** Compared to the healthy controls, the ANR group had significantly lower visual learning and social cognition scores, and the ANBP group had significantly lower processing speed, attention/vigilance, visual learning, reasoning/problem-solving, and social cognition scores. Compared to the ANR group, the ANBP group had significantly lower attention/vigilance scores.

**Discussion:** The AN subtypes differed in cognitive function impairments. Participants with ANBP, which is associated with higher mortality rates than ANR, exhibited greater impairment severities, especially in the attention/vigilance domain, confirming the presence of impairments in continuous concentration. This may relate to the impulsivity, an ANBP characteristic reported in the personality research. Future studies can further clarify the cognitive impairments of each subtype by addressing the subtype cognitive functions and personality characteristics.

## Introduction

Anorexia nervosa (AN) is a disease characterized by extreme anxiety about eating, a pursuit of weight loss, and a distorted body image [1]. Its mortality rate is exceedingly high, even when compared to psychiatric diseases that are generally chronic [2]. Although AN is known to be associated with both biological and psychosocial factors, its etiology is poorly understood, and no effective treatment is yet available. Recent studies have implicated cognitive dysfunctions in the development and maintenance of AN [3], and researchers are increasingly interested in cognitive functioning in AN, including aspects such as set-shifting [4–7], central coherence [8, 9], visuospatial abilities [10], and decision-making [11, 12]. Of these aspects, consistent findings have emerged for set-shifting and central coherence [4–9]. Set-shifting is related to flexibility in task performance; therefore, set-shifting impairments hinder adaptation to unfamiliar situations. Weak central coherence, which is believed to induce an excessive focus on details at the expense of big-picture thinking, is reportedly a characteristic cognitive dysfunction in autism spectrum disorder [13]. The weak central coherence in AN is more pronounced in visuospatial tasks than in verbal tasks [4], and these cognitive dysfunctions are reportedly closely tied to core AN symptoms such as the morbid pursuit of thinness and body image distortions [8]. In relation to body image impairment, it has been suggested that compulsively repeated body checking may reinforce negative perception, resulting in distorted beliefs of body image [14]. One of the factors that can lead to increased number of body checking behaviors is body dissatisfaction, which is conceptualized as a multi-dimensional construct consisting of behaviors, cognition and affect; it has been reported to be a candidate of a risk factor for AN onset [15].

Cognitive dysfunctions in AN also affect social adaptation and interpersonal relationships, and this has further consequences for functional outcomes [16]. Cognitive dysfunctions in AN are therefore believed to be associated with AN's core symptoms and patients' social functioning.

AN manifests in a restricting (ANR) subtype, in which patients limit food consumption, and a binge-eating/purging (ANBP) subtype, in which patients exhibit cycles of large meals followed by purging behaviors. Both subtypes share core clinical symptoms including efforts to maintain abnormally low weight, a fear of obesity, and body image disturbances, but there are clear personality and behavioral differences between persons with ANR and those with ANBP [17–20].

As for cognitive functions, past subtype-comparison studies have reported conflicting results. Although the studies agree that weak central coherence and poor set-shifting are commonly found in AN generally, no agreement has been reached in terms of the severity differences of these cognitive domains between the subtypes [21–23]. Furthermore, there is no consensus about dysfunctions in other cognitive domains in AN subtypes [24]. One of the reasons could be that there has been no study to our knowledge that comprehensively evaluated the separable cognitive functions with uniform and standardized test batteries. It would be extremely important to use the consensus assessment batteries because the preceding studies on AN subtype differences in cognitive functions used different tests to evaluate the same cognitive domain, resulting in inconsistent interpretation of the findings. For example, Rose et al. used the Ravello Profile, a cognitive function assessment battery for eating disorders, which can evaluate domains such as performance IQ, Verbal IQ, Visuospatial Memory, Visuospatial Processing, Verbal Fluency, Executive Functioning [25]. However, it cannot evaluate cognitive domains yet to be shown as impaired since it includes only those scales related to cognitive dysfunctions that are considered specific to eating disorders.

Therefore, for this study, we chose to use the MATRICS Consensus Cognition Battery (MCCB), which was originally designed to evaluate cognitive functions in patients with schizophrenia [26–28] and is appropriate for comprehensively assessing basic cognitive functions in order to characterize the extensive cognitive domains of AN subtypes. Because patients frequently alternate between the ANR and ANBP subtypes [29], elucidating the neuropsychological differences and similarities between the subtypes may clarify the pathophysiology of AN.

We developed a Japanese version of the MCCB (MCCB-J) and confirmed its validity and reliability for Japanese patients with schizophrenia [30] and its utility for detecting cognitive dysfunctions in Japanese patients with bipolar disorder [31]. The MCCB has been used to study mental illnesses other than schizophrenia, such as posttraumatic stress disorder [32] and treatment-resistant depression [33], and it has been used to identify cognitive dysfunctions in many other disorders [34, 35]. Although a previous MCCB-based study of AN found no cognitive impairments [36], we aimed to comprehensively examine the neurocognitive features and cognitive functions in each AN subtype using the MCCB-J.

## Materials and methods

### Participants and procedures

We consecutively recruited female outpatients or inpatients with AN at the Kobe University Hospital with a targeted age range of 15–60 years. An experienced psychiatrist confirmed AN diagnoses through the clinical interview and we included patients in partial remission who fulfilled all of the diagnostic criteria except for a sustained period of low body weight. The exclusion criteria included a history of drug or alcohol abuse, a comorbid psychopathology related to drug or alcohol abuse, imminent suicidality, any indication of severe mental illness necessitating inpatient treatment, any serious medical condition, a serious daily living impairment due to psychiatric symptoms, or an IQ below 80 as assessed on the Japanese Adult Reading Test (JART) [37]. JART is the Japanese version of the National Adult Reading Test (NART) that was developed to estimate IQ in native English-speaking patients, and its validity and reliability have been confirmed [38]. The presence or absence of illnesses in the exclusion criteria was checked by asking about current psychopathology and developmental history in the clinical interview and by reviewing the past medical records. No recruited subjects were excluded from the analyses based on these criteria or refused to participate. Forty participants met the diagnostic criteria for AN in the Diagnostic and Statistical Manual of Mental Disorders, 5th Edition [1], and did not meet any exclusion criteria. Twenty-two patients (8 inpatients and 14 outpatients) exhibited the ANR subtype (mean age 27.59 ± 11.96, mean BMI14.27 ± 2.68, mean level of education 13.36 ± 2.20) and 18 patients (8 inpatients and 10 outpatients) exhibited the ANBP subtype (mean age 30.61 ± 11.97, mean BMI16.79 ± 2.69, mean level of education 12.83 ± 1.95). The main diagnosis was either ANR or ANBP and there was no comorbidity including another subtype of AN. Medications were taken by nine of ANBP (antidepressant, *n* = 3, antipsychotic, *n* = 5, benzodiazepine, *n* = 1) and six of ANR (antidepressant, *n* = 2, antipsychotic, *n* = 2, benzodiazepine, *n* = 2) at the time of assessment. The data were collected for two years between June, 2015 and June, 2017.

For healthy controls, we recruited 69 female community residents with ages between 15 and 60 years inclusive and no histories of any eating disorders or any other psychiatric disorders through personal contact and public advertisement in the local community. Demographic data for patients and healthy controls are summarized in Table 1.

**Table 1:** Demographic and clinical characteristics of the subjects.

	**ANR group (*n* = 19–21)**	**ANBP group (*n* = 16–18)**	**Healthy controls (*n* = 69)**	**Group comparisons^a^**		***Post-hoc* comparisons**
		**Mean ± SD**		**Statistics**	***p*-value**	
Age (years)	27.59 ± 11.96	30.61 ± 11.97	34.36 ± 11.03	*F* = 3.22	0.044	ANR < HC(*p* = 0.044)
Estimated IQ^b^	101.67 ± 9.02	100.53 ± 9.95	106.10 ± 8.50	*F* = 3.93	0.023	n. s.
Education (years)	13.36 ± 2.20	12.83 ± 1.95	15.26 ± 2.27	*F* = 12.18	<0.001	ANR < HC(*p* = 0.002)
						ANBP < HC(*p* < 0.001)
Chart-recorded minimum BMI (kg/m^2^)	11.54 ± 1.98	12.92 ± 1.99	–	*F* = 4.35	0.045	ANR < ANBP
BMI at assessment (kg/m^2^)	14.27 ± 2.68	16.79 ± 2.69	–	*F* = 7.97	0.008	ANR < ANBP
Illness duration (years)	9.29 ± 7.21	10.35 ± 7.24	–	*F* = 0.19	0.662	n. s.
EDE-Q total	2.06 ± 1.36	2.43 ± 1.37	–	*F* = 0.60	0.446	n. s.

*ANR, anorexia nervosa, restricting subtype; ANBP, anorexia nervosa, binge-eating/purging subtype; IQ, intelligence quotient; BMI, body mass index; EDE-Q, Eating Disorder Examination-Questionnaire; SD, standard deviation; ns, not significant*.

a *Group comparisons; One–way analysis of variance for age, estimated IQ, and education. Analyses of covariance for chart-recorded minimum BMI, BMI at assessment, illness duration and EDE-Q total score*.

b *Estimated IQ, One-way analysis of variance revealed significant between-group differences, but the post-hoc Tukey's test revealed no such differences*.

Written consent was obtained from all participants. We also obtained the written informed parental consent for participants under the age 16. The study was conducted according to the standards of the Declaration of Helsinki and was approved by the Kobe University Hospital Ethics Committee.

### Measures

AN severity was assessed using the Eating Disorder Examination-Questionnaire (EDE-Q) [39, 40]. As alluded to, each participant's IQ was measured with the JART, which is the validated Japanese version of the NART [37]. As mentioned, our neurocognitive assessments were based on the MCCB-J [30], which was administered by clinical psychologists who had completed MCCB-J training. The MCCB-J consists of 10 subtests that assess seven cognitive domains [41], including (1) processing speed, which is assessed using the Trail Making Test, part A (TMT-A), the Brief Assessment of Cognition in Schizophrenia–Symbol Coding test (BACS-SC), and the Category Fluency–Animal Naming test; (2) attention/vigilance, which is assessed with the Continuous Performance Test–Identical Pairs (CPT-IP); (3) working memory, which is assessed using the University of Maryland–Letter-Number Span test (LNS), and the Wechsler Memory Scale III Spatial Span test (WMS-SS); (4) verbal learning, which is assessed using the Hopkins Verbal Learning Test–Revised (HVLT–R); (5) visual learning, which is assessed using the Brief Visuospatial Memory Test–Revised (BVMT–R); (6) reasoning/problem-solving, which is assessed using the Neuropsychological Assessment Battery–Mazes (NAB); and (7) social cognition, which is assessed using the Mayer-Salovey-Caruso Emotional Intelligence Test's Managing Emotions component (MSCEIT-ME). Each participant completed the full MCCB-J during one session that took ~90 min.

### Statistical analysis

Because our participants were all Japanese, we did not use the published MCCB normative data as reference data [42]. Instead, we computed T-scores from the means and standard deviations (SDs) of the Japanese normative data derived from the age-corrected standard scores from the MCCB scoring program [42]. The normative data for the MCCB-J are based on 202 participants from six Japanese cities. For all further analyses, we used data from our healthy controls as reference data.

We used one-way analysis of variance (ANOVA) to compare the ANR, ANBP, and healthy control groups for demographic and clinical characteristics. We then conducted *post-hoc* pairwise multiple comparisons corrections for significant differences with Tukey's test. We used analyses of covariance (ANCOVA) to compare the ANR and ANBP groups for chart-recorded minimum body mass indices (BMIs), BMIs at assessment, illness durations and EDE-Q controlling for three demographic variables (i.e., IQ, age, and years of education) as covariance.

For between-group comparisons of MCCB-J scores, we conducted a multivariate analysis of covariance (MANCOVA) with the seven MCCB-J domain T-scores as the dependent variables, the three groups as the subject variables, and the three demographic variables exhibiting significant between-group differences (i.e., IQ, age, and years of education) as covariates. We then applied Bonferroni multiple comparisons corrections for significant differences.

For the ANR and ANBP groups, we calculated partial correlation coefficients with the three demographic variables (i.e., IQ, age, and years of education) as control variables to determine how the chart-recorded minimum BMIs, BMIs at assessment, and illness durations correlated with MCCB-J neurocognitive performance scores.

All statistical analyses were conducted with SPSS version 12.0 (IBM, Armonk, NY). Statistical significance was defined as *p* < 0.05.

## Results

### Clinical and demographic features

Table 1 displays the means and SDs for the three groups' demographic and clinical characteristics. The ANOVA revealed significant between-group differences in age [*F*_(2, 106)_ = 3.22, *p* = 0.044], education level [*F*_(2, 106)_ = 12.18, *p* < 0.001], and IQ [*F*_(2, 104)_ = 3.93, *p* = 0.023]. *Post-hoc* application of Tukey's test showed that the ANR group was significantly younger than the healthy controls (*p* = 0.044), but the ANBP group did not significantly differ in age from the healthy controls (*p* = 0.43) or the ANR group (*p* = 0.68). *Post-hoc* analysis of educational levels revealed that compared to the healthy controls, the ANR (*p* = 0.002) and ANBP (*p* < 0.001) groups had significantly fewer years of education. However, it revealed no significant difference between the ANR and ANBP groups (*p* = 0.73). In terms of IQ, *post-hoc* testing revealed no significant differences between the ANR group and the healthy controls (*p* = 0.11), between the ANBP group and the healthy controls (*p* = 0.055), or between the ANR and ANBP groups (*p* = 0.92).

Compared to the ANBP group, the ANR group exhibited significantly lower minimum chart-recorded BMIs (*F* = 4.35, *p* = 0.045) and BMIs at assessment (*F* = 7.97, *p* = 0.008). However, the two groups did not significantly differ in illness durations (*F* = 0.19, *p* = 0.662) or EDE-Q scores (*F* = 0.60, *p* = 0.446).

### Mccb-j neurocognitive function scores

**Figure 1:**
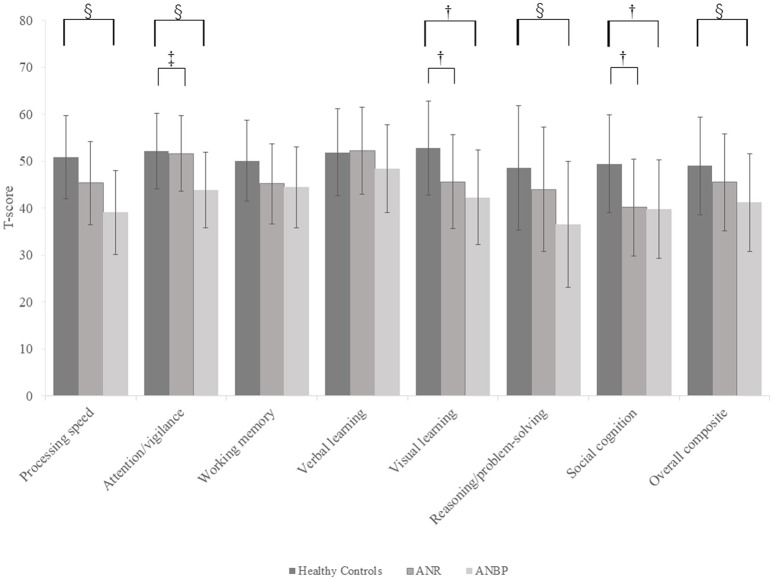
Multivariate analysis of covariance for all MCCB-J domains and overall cognitive composite T-scores of the subjects. Mean T-scores for all MCCB-J domains and overall cognitive composite scores for the subjects. Error bars show standard deviation. ANBP, anorexia nervosa, binge-eating/purging subtype; ANR, anorexia nervosa, restricting subtype; MCCB-J, MATRICS Consensus Cognitive Battery, Japanese-language version. ^†^Significant pairwise differences between the healthy controls and both the ANBP and ANR groups (*p* < 0.05).^‡^Significant pairwise difference between the ANBP and ANR groups (*p* = 0.009). ^§^Significant pairwise difference between the ANBP group and the healthy controls (*p* < 0.005).

Figure 1 and Supplementary Table 1 show the mean T-score profiles for the MCCB-J domains in the ANR, ANBP, and healthy control groups. The MANCOVA of MCCB-J domain scores revealed a significant overall group effect for neurocognitive domain performance [F_(14, 190)_ = 3.617, *p* < 0.001, Wilk's lambda = 0.623]. When domain-specific results were considered, we found significant group effects for the processing speed, attention/vigilance, visual learning, reasoning/problem-solving, and social cognition domains. These results survived the Bonferroni correction. *Post-hoc* comparisons to the healthy controls revealed that the ANR group scored significantly lower in the visual learning (*p* = 0.019) and social cognition (*p* = 0.002) domains and that the ANBP group scored significantly lower in the processing speed (*p* < 0.001), attention/vigilance (*p* = 0.001), visual learning (*p* = 0.001), reasoning/problem-solving (*p* = 0.005), and social cognition (*p* = 0.004) domains. Compared to the ANR group, the ANBP group scored significantly lower in the attention/vigilance domain (*p* = 0.009).

**Figure 2:**
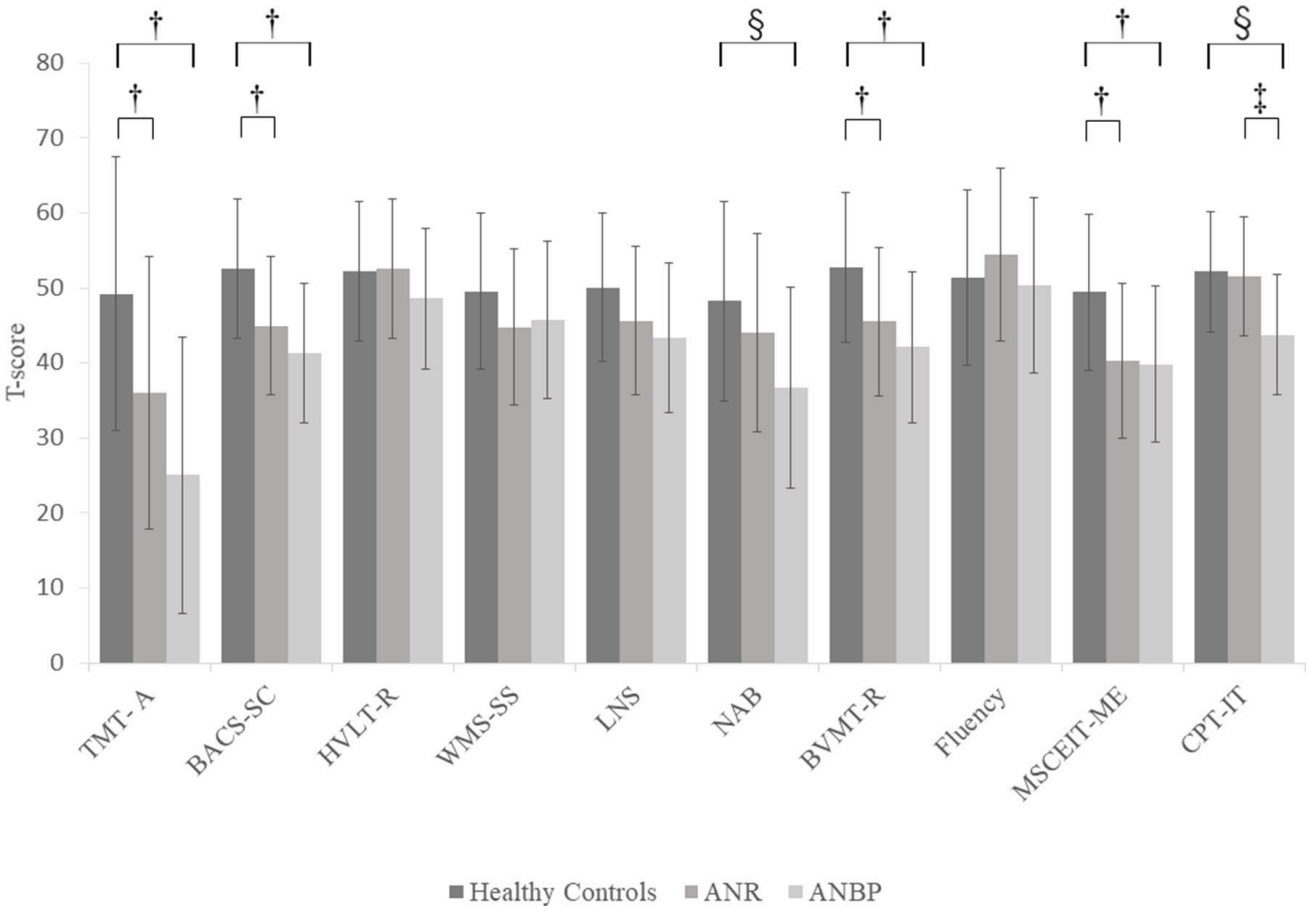
Multivariate analysis of covariance for MCCB-J subtest T-scores of the subjects. Mean T-scores for all MCCB-J subtest scores for the subjects. Error bars show standard deviation. ANBP, anorexia nervosa, binge-eating/purging type subtype; ANR, anorexia nervosa, restricting subtype; BACS-SC, Brief Assessment of Cognition in Schizophrenia–Symbol Coding test; BVMT-R, Brief Visuospatial Memory Test-Revised; CPT-IP, Continuous Performance Test–Identical Pairs; Fluency, Category Fluency–Animal Naming test; HVLT-R, Hopkins Verbal Learning Test-Revised; LNS, University of Maryland–Letter-Number Span test; MCCB, MATRICS Consensus Cognitive Battery, Japanese-language version; MSCEIT-ME, Mayer-Salovey-Caruso Emotional Intelligence Test, Managing Emotions component; NAB, Neuropsychological Assessment Battery–Mazes; SD, standard deviation; TMT-A, Trail Making Test, part A; WMS-SS, Wechsler Memory Scale III Spatial Span test. ^†^Significant pairwise differences between the healthy controls and both the ANBP and ANR groups (*p* < 0.05).^‡^Significant pairwise difference between the ANBP and ANR groups (*p* = 0.008). ^§^Significant pairwise difference between the ANBP group and the healthy controls (*p* < 0.01).

Figure 2 and Supplementary Table 2 show the mean T-scores of the MCCB-J subtests for the three groups. The MANCOVA showed a significant overall group effect [*F*_(20.184)_ = 3.043, *p* < 0.001, Wilk's lambda = 0.565]. Compared to the healthy controls, the ANR and ANBP groups scored significantly lower on the TMT-A, BACS-SC, LNS, NAB, BVMT-R, MSCEIT-ME, and CPT-IP subtests, but the significant group effects for the LNS and NAB subtests disappeared after Bonferroni corrections. *Post-hoc* comparisons with the healthy controls showed that the ANR group scored significantly lower on the TMT-A (*p* = 0.017), BACS-SC (*p* = 0.006), BVMT-R (*p* = 0.018), and MSCEIT-ME (*p* = 0.003) subtests and that the ANBP group scored significantly lower on the TMT-A (*p* < 0.001), BACS-SC (*p* < 0.001), NAB (*p* = 0.007), BVMT-R (*p* = 0.001), MSCEIT-ME (*p* = 0.004), and CPT-IP (*p* = 0.001) subtests. Compared to the ANR group, the ANBP group scored significantly lower on the CPT-IP subtest (*p* = 0.008).

### Correlations between clinical characteristics and neurocognitive functioning scores

As shown in Supplementary Table 3 and Supplementary Figure 1, MCCB-J neurocognitive performance scores did not correlate with chart-recorded minimum BMIs (ANR group: −0.361 ≤ *r* ≤ 0.082, ANBP group: −0.407 ≤ *r* ≤ 0.269), BMIs at assessment (ANR group: −0.197 ≤ *r* ≤ 0.303, ANBP group: −0.343 ≤ *r* ≤ 0.358), or illness durations (ANR group: −0.270 ≤ *r* ≤ 0.290, ANBP group: −0.112 ≤ *r* ≤ 0.507).

## Discussion

We aimed to comprehensively examine the cognitive characteristics of patients with the AN subtypes ANR and ANBP by using the MCCB-J, a comprehensive cognitive assessment for Japanese patients with schizophrenia, to systematically compare cognitive functions in patients with either subtype to those in healthy controls.

We found that compared to the healthy controls, both patient groups scored significantly lower in the visual learning and social cognition domains, with the ANBP group also scoring significantly lower in the processing speed, attention/vigilance, and reasoning/problem-solving domains. Furthermore, compared to the ANR group, the ANBP group scored significantly lower in the attention/vigilance domain. However, the patient groups and healthy controls achieved similar scores in the verbal learning and working memory domains. These results clearly characterized the cognitive dysfunctions of each AN subtype. Furthermore, we found no statistically significant correlations between the cognitive variables and BMIs or illness durations, which suggests that emaciation does not affect the cognitive variables.

The MCCB, which assesses seven cognitive domains with 10 subtests that have superb tolerability, practicality, and test-retest reliability, can be used repeatedly [41]. Another specific quality of the MCCB is its co-norming with a healthy population for standardization [42]. The seven cognitive domains were chosen because (1) they are potential targets for novel schizophrenia treatments, (2) they were examined in many past studies on cognitive dysfunctions in schizophrenia [43], and (3) they were separable neurocognitive factors previously examined in healthy controls using the Wechsler Adult Intelligence Scale III and Wechsler Memory Scale III [44]. Thus, the MCCB defines separable neurocognitive domains from healthy control data and incorporates the cognitive characteristics of schizophrenia.

The processing speed domain was assessed with the TMT-A and BACS-SC, which both measure processing speed through non-verbal domains, and a category fluency test, which assesses it through verbal domains. Both patient groups scored significantly lower on the TMT-A and BACS-SC than the healthy controls did, but there were no significant differences in category fluency scores. The TMT-A scores in particular were the lowest subtest scores for both AN groups. The TMT is among the most frequently used assessment tools for set-shifting [5–7, 17, 21, 23], which is characteristic of AN-associated cognitive dysfunctions. The TMT consists of part A, in which subjects serially connect numbers, and part B, in which subjects serially connect numbers and letters in turn. Although only the TMT-A is incorporated into the MCCB, the low TMT-A scores, which reflect TMT-B scores [45], suggest that cognitive flexibility is impaired in AN. Another characteristic of both AN groups in the processing speed domain was that non-verbal processing was slow whereas verbal processing was normal. This implies that although verbal information can be processed normally, visual information processing is problematic. As for overall processing speed domain scores, only the ANBP group scored significantly lower than the healthy controls because the ANBP group's TMT-A and BACS-SC scores were extremely low when compared to those of the healthy controls.

We evaluated the attention/vigilance domain with the CPT-IP, in which subjects press a button when identical numbers appear on a computer screen. This test measures sustained attention. The ANR group's CPT-IP scores were similar to those of the healthy controls, which suggests the absence of serious attention-arousal problems. However, the ANBP group scored significantly lower than both the healthy controls and the ANR group, which suggests that continuous concentration is impaired in ANBP. This represents the first report of CPT-IP-measured differences in continuous concentration between the ANR and ANBP subtypes. It should be emphasized that attention/vigilance was the only MCCB-J cognitive function domain for which we found a significant difference between the subtypes.

In the reasoning/problem-solving domain, we again found that the ANBP group scored significantly lower than the healthy controls did whereas the ANR group did not. We evaluated reasoning/problem-solving abilities with the NAB, which uses drawn mazes to assess insight and planning abilities that are related to conceptual understanding and objective observation capacities. Since attention and concentration are related to these conceptual activities [45], the ANBP group scored significantly lower in this domain than the healthy controls did, as was the case for the attention/vigilance domain. These results suggest that patients with ANBP experience difficulties in organization and planning.

For the working memory domain, we used the WMS-SS for non-verbal working memory and the LNS for verbal working memory. On the WMS-SS, neither patient group scored significantly lower than the healthy controls did. There were also no significant differences on the LNS after Bonferroni corrections or in pairwise comparisons of the healthy controls with either patient group. Therefore, in this study, working memory was intact in both AN subtypes.

We assessed the verbal learning domain with the HVLT-R and found that neither patient group significantly differed from the healthy controls. The ANR group in particular scored similarly to the healthy controls. Together with the fact that the ANR group scored higher than the healthy controls in the category fluency test, which reflects verbal processing speed, this implies that verbal domains are not impaired in the ANR subtype. Our results are consistent with those of a previous report [46] that language domain performance in patients with AN is no different from, and sometimes superior to, that of healthy controls.

We assessed visual learning with the BVMT-R, on which both patient groups scored significantly lower than the healthy controls did. These results confirm those of previous studies [10, 47] that reported impaired visual perception and visuospatial abilities in both AN subtypes. Visuospatial impairments and weak central coherence at the visuospatial level were the most frequently targeted impairments in the past AN-related studies, and those studies reported that these cognitive dysfunctions affect AN's onset and duration [4]. Of the available visuospatial domain measures, the Rey-Osterrieth Complex Figure Test (RCFT) [48], in which subjects copy a complex figure and later reproduce it from memory, has been the most commonly employed and is included in the Ravello Profile [25] that serves as a cognitive function battery for patients with AN. Although the BVMT-R that is included in the MCCB utilizes a simpler figure than the RCFT does, both patient groups scored significantly lower than the healthy controls did. This finding confirms the visuospatial memory and cognition impairments of both AN subtypes as reported in previous studies [10, 47] and further implies the seriousness of these impairments since lower scores were obtained even with the BVMT-R's relatively simple test.

Cognitive function domains can be classified into neurocognitive domain and social cognitive domain. Social cognition consists of mental processes that underlie social interactions and is defined as the ability to perceive others' intentions and internal states [49]. The impairment of social cognition is reported to have a close relationship with daily living functions, and also associated with functional outcome [50]. In our current study, both ANR and ANBP were impaired in the social cognitive domain. Interestingly, although the ANR group's impairments in some neurocognitive domains were milder than those of the ANBP group, both groups exhibited similarly low social cognition domain scores. This finding may mean that ANR has comparable impairments in daily living functions as ANBP. AN-related social cognition impairments have been widely studied, and previous studies reported impairments of facial cognition [51] and theory of mind [52, 53, 54]. The MSCEIT-ME, which measures emotional control in conflictual situations, revealed that both AN groups had problems with such emotional control. Our results suggest that both AN subtypes have social cognition domain impairments, as previously reported, and that these impairments specifically affect emotional control in conflictual situations.

As noted, our ANBP group exhibited broader cognitive function impairments than our ANR group did. Our study is the first to report this subtype-specific difference in cognitive dysfunction severities. Clinical experiences also suggest that patients with ANBP more frequently exhibit kleptomania, substance dependence, suicide, and self-mutilation, which are all related to the impulsivity often observed in ANBP [17, 55], as well as comorbid depression and mood lability [56]. A longitudinal study also reported lower remission rates and higher mortality rates for patients with ANBP and poor prognoses [54]. The broader cognitive impairments of ANBP observed in our study are consistent with these clinical features, and, conversely, more severe cognitive impairments may be related to these features.

Our results reinforce previous reports that cognitive impairments in AN do not correlate with BMIs or illness durations [6, 7, 21, 47, 57, 58]. It is reported that cognitive impairment could be a marker of chronicity in AN or a risk indicator for the development of chronic AN [59]. Furthermore, set-shifting impairments, which have been observed in unaffected sisters of AN probands [21] and in patients with AN who recovered to normal weight [4, 60], could be an endophenotype [61]. This suggests that the cognitive impairments seen in AN may be traits unrelated to ill state.

Phillipou et al.'s study [36] is the only previous one to our knowledge that examined cognitive functions in AN with the MCCB, and it revealed that relative to healthy controls, the patients had significantly delayed false alarm responses on the CPT-IP and significantly different scores on the WMS-SS backward component but no significant differences in cognitive domain scores or subtest scores. But the authors noted that overall cognitive functioning was unimpaired in AN with the MCCB. These findings differ from ours, probably due to differences in the subjects. Phillipou et al. selected subjects who were medically stable but had suboptimal BMIs to minimize the influence of malnutrition on their results. Although our subjects were medically stable and had an average BMI comparable to that of Phillipou et al.'s subjects, our subjects had a greater average age and longer average illness duration. Also our subjects were distinctive in terms of its very low BMIs compared to previous studies [21, 60]; chart-recorded minimum BMIs of ANR being 11.54 ± 1.98 (kg/m^2^) and that of ANBP 12.92 ± 1.99 (kg/m^2^). ANBP showed higher mean EDE-Q scores than ANR, but this was not statistically significant. The reason could be that the EDE-Q scores may not necessary corresponds to the severity. As for relationship between illness severity and cognitive function, Phillipou et al.'s milder cases showed no significant cognitive impairment relative to healthy controls. The current study, which included more severe cases, showed cognitive domains with significantly lower cognitive functioning. These findings may suggest that cognitive function of our subjects was more impaired because the illness was more severe with very low chart-recorded minimum BMIs. Furthermore, cognitive impairments in the ANR group were milder than in the ANBP group in the current study, despite the ANR group having significantly lower minimum chart-recorded BMIs and BMIs at assessment. It is therefore unlikely that malnutrition directly relates to cognitive impairment, so just as a previous study [59] suggested that cognitive impairments are a risk factor for chronicity.

Any contribution of comorbid conditions such as depression and anxiety need to be discussed as well. Our current study could not examine the influence of comorbidity since there were no cases with comorbid depressive disorders or anxiety disorders. According to the data of the preceding studies [9, 5], neuropsychological performance did not correlate with level of anxiety and depression, which suggests that comorbid symptoms such as depression and anxiety may not influence cognitive functions.

This study has some limitations. It was a cross-sectional study, so it could not capture the whole picture of AN. Future prospective and longitudinal studies might provide more in-depth findings about subtype-specific cognitive impairments. Another limitation was that the sample size was relatively small. Future studies with larger samples are needed to validate our findings.

In summary, we found that MCCB-J scores for the visual learning and social cognition domains were significantly lower in both AN subtypes. Furthermore, the ANBP group scored lower than the ANR group did in all MCCB-J cognitive domains, which indicates broader cognitive impairments in ANBP. It was especially notable that we observed a difference in the attention/vigilance domain. This may relate to the impulsivity, an ANBP characteristic reported in the personality research [17–20]. Future studies may clarify the factors that contribute the development of eating disorders by examining the relationship between cognitive functions and psychological profile of ANR and ANBP including perfectionism characteristic to ANR. As this is the first systematic study of the previously unclear subtype-specific differences in cognitive impairments in AN, our results may be extremely valuable for future efforts to design treatment strategies and elucidate the pathophysiology of AN. We expect that targeting the cognitive profile characteristics observed in our study will prevent severe and enduring AN and enhance improvements in social functioning.

## Author contributions

HT and IS designed the study. HT, YK, and IS collected the data. AO administered the psychological tests. YA and SM analyzed the data. HT, RC and AO wrote the draft. HT and IS wrote the final manuscript. All authors approved the final manuscript.

## Conflict of interest statement

The authors declare that the research was conducted in the absence of any commercial or financial relationships that could be construed as a potential conflict of interest. The reviewer YH and handling Editor declared their shared affiliation.
