# Supplementary material for: Neurocognitive Impairments Are More Severe in the Binge-Eating/Purging Anorexia Nervosa Subtype Than in the Restricting Subtype
Source: Front Psychiatry. 2018 Apr 16;9:138. doi: 10.3389/fpsyt.2018.00138 (PMC5911723; doi:10.3389/fpsyt.2018.00138)
Supplement: Supplementary file 1 [file Table1.pdf]

## Supplementary Material:

# Neurocognitive Impairments are More Severe in the Binge-eating/Purging Anorexia Nervosa Subtype than in the Restricting Subtype

**Hiroko Tamiya, MD[1], Atushi Ouchi, MA[1], Runshu Chen, MD[1], Shiho Miyazawa, PhD[2], Yoritaka Akimoto, PhD[3], Yasuhiro Kaneda, MD, PhD[4], Ichiro Sora, MD, PhD[1*]**

[1]Department of Psychiatry, Kobe University Graduate School of Medicine, Kobe, Japan

[2]Department of Biological Psychiatry, Tohoku University Graduate School of Medicine, Sendai, Japan

[3]Department of Information & Management Systems Engineering, Nagaoka University of Technology, Nagaoka, Japan

[4]Department of Psychiatry, Iwaki Clinic, Tokushima, Japan

*** Correspondence:** Ichiro Sora, MD, PhD: sora@med.kobe-u.ac.jp

**Supplementary Table 1. Multivariate analysis of covariance for all MCCB-J domains and overall cognitive composite T-scores of the sample**

| MCCB-J domains | ANR group (n = 21) | ANBP group (n = 17) | Healthy controls (n = 69) | $F$ [a] | $p$-value | partial $\eta^2$ | Post hoc comparisons |
|---|---|---|---|---|---|---|---|
| | | Mean ± SD | | | | | |
| **Processing speed** | 45.35±8.87 | 39.06±8.96 | 50.88±8.91 | 11.69 | <0.001 | 0.19 | ANBP <HC (p<0.001) |
| **Attention/vigilance** | 51.63±8.00 | 43.89±8.08 | 52.18±8.03 | 7.18 | 0.001 | 0.13 | ANBP<ANR(p=0.009)<br>ANBP <HC (p=0.001) |
| **Working memory** | 45.17±8.53 | 44.48±8.62 | 50.09±8.57 | 3.99 | 0.021 | 0.07 | n. s. |
| **Verbal learning** | 52.27±9.26 | 48.38±9.35 | 51.90±9.30 | 1.09 | 0.339 | 0.02 | n. s. |
| **Visual learning** | 45.62±10.02 | 42.29±10.12 | 52.80±10.07 | 8.40 | <0.001 | 0.14 | ANR < HC (P=0.019)<br>ANBP <HC (P=0.001) |
| **Reasoning/problem-solving** | 44.00±13.23 | 36.56±13.37 | 48.60±13.30 | 5.28 | 0.007 | 0.10 | ANBP <HC (p=0.005) |
| **Social cognition** | 40.19±10.32 | 39.77±10.43 | 49.44±10.37 | 8.85 | <0.001 | 0.15 | ANR < HC (p=0.002)<br>ANBP< HC (p=0,004) |
| **Overall composite score** | 45.51±10.32 | 41.19±10.42 | 49.00±10.37 | 3.74 | 0.027 | 0.07 | ANBP < HC (p=0.025) |

Abbreviations: ANBP, anorexia nervosa, binge-eating/purging subtype; ANR, anorexia nervosa, restricting subtype; MCCB-J, MATRICS Consensus Cognitive Battery, Japanese-language version; n. s., not significant.
