# Supplementary material for: Neurocognitive Impairments Are More Severe in the Binge-Eating/Purging Anorexia Nervosa Subtype Than in the Restricting Subtype
Source: Front Psychiatry. 2018 Apr 16;9:138. doi: 10.3389/fpsyt.2018.00138 (PMC5911723; doi:10.3389/fpsyt.2018.00138)
Supplement: Supplementary file 2 [file Table2.pdf]

**Supplementary table 2. Multivariate analysis of covariance for MCCB-J subtest T-scores of the sample**

| MCCB-J subtest | ANR group (n = 21) | ANBP group (n = 17) | Healthy controls (n = 69) | *F* [a] | *p*-value | partial $\eta^2$ | Post hoc comparisons |
|---|---|---|---|---|---|---|---|
| | | Mean ± SD | | | | | |
| **TMT- A** | 35.98±18.19 | 25.01±18.37 | 49.24±18.27 | 12.21 | <0.001 | 0.20 | ANBP <HC(p<0.001)<br>ANR<HC(p=0.017) |
| **BACS-SC** | 44.96±9.24 | 41.36±9.33 | 52.54±9.29 | 11.15 | <0.001 | 0.18 | ANBP<HC(p<0.001)<br>ANR<HC(p=0.006) |
| **HVLT-R** | 52.49±9.30 | 48.60±9.40 | 52.23±9.34 | 1.12 | 0.33 | 0.02 | n. s. |
| **WMS-SS** | 44.76±10.39 | 45.77±10.50 | 49.58±10.44 | 1.93 | 0.15 | 0.04 | n. s. |
| **LNS** | 45.64±9.85 | 43.36±9.95 | 50.02±9.90 | 3.41 | 0.037 | 0.06 | n. s. |
| **NAB** | 43.96±13.22 | 36.70±13.35 | 48.25±13.28 | 4.86 | 0.010 | 0.09 | ANBP<HC(p=0.007) |
| **BVMT-R** | 45.49±9.94 | 42.09±10.04 | 52.67±9.99 | 8.61 | <0.001 | 0.15 | ANBP<HC(p=0.001)<br>ANR<HC(p=0.018) |
| **Fluency** | 54.46±11.59 | 50.37±11.71 | 51.38±11.65 | 0.75 | 0.475 | 0.02 | n. s. |
| **MSCEIT-ME** | 40.31±10.34 | 39.85±10.45 | 49.45±10.39 | 8.64 | <0.001 | 0.15 | ANBP<HC(p=0.004)<br>ANR<HC(p=0.003) |
| **CPT-IT** | 51.53±7.93 | 43.77±8.01 | 52.16±7.97 | 7.46 | 0.001 | 0.13 | ANBP<HC(p=0.001)<br>ANBP<ANR(p=0.008) |

Abbreviations: ANBP, anorexia nervosa, binge-eating/purging type subtype; ANR, anorexia nervosa, restricting subtype; BACS-SC, Brief Assessment of Cognition in Schizophrenia–Symbol Coding test; BVMT-R, Brief Visuospatial Memory Test-Revised; CPT-IP, Continuous Performance Test–Identical Pairs; Fluency, Category Fluency–Animal Naming test; HVLT-R, Hopkins Verbal Learning Test-Revised; LNS, University of Maryland–Letter-Number Span test; MCCB, MATRICS Consensus Cognitive Battery, Japanese-language version; MSCEIT-ME, Mayer-Salovey-Caruso Emotional Intelligence Test, Managing Emotions component; NAB, Neuropsychological Assessment Battery–Mazes; SD, standard deviation; TMT-A, Trail Making Test, part A; WMS-SS, Wechsler Memory Scale III Spatial Span test; n. s., not significant.

[a] Multivariate analysis of covariance controlling for age, IQ, and educational level.
