# Supplementary material for: Neurocognitive Impairments Are More Severe in the Binge-Eating/Purging Anorexia Nervosa Subtype Than in the Restricting Subtype
Source: Front Psychiatry. 2018 Apr 16;9:138. doi: 10.3389/fpsyt.2018.00138 (PMC5911723; doi:10.3389/fpsyt.2018.00138)
Supplement: Supplementary file 3 [file Table3.pdf]

Supplementary table 3. Partial correlations of MCCB-J domain scores with chart-recorded minimum BMIs, BMIs at assessment, and illness durations with the three demographic variables (i.e., IQ, age, and years of education) as control variables

		ANR group			ANBP group		
**Cognitive domains**		Chart-recorded minimum BMIs	BMIs at assessment	Illness duration	Chart-recorded minimum BMIs	BMIs at assessment	Illness duration
**Processing speed**	*r*	-0.032	0.049	-0.270	0.042	0.252	0.239
	p	0.899	0.846	0.279	0.892	0.406	0.431
**Attention/vigilance**	*r*	-0.099	-0.197	0.034	-0.407	-0.038	0.232
	p	0.697	0.434	0.894	0.168	0.903	0.445
**Working memory**	*r*	0.082	0.162	0.021	0.269	0.358	-0.112
	p	0.748	0.520	0.935	0.374	0.230	0.717
**Verbal learning**	*r*	-0.113	0.043	0.109	-0.085	0.059	0.331
	p	0.655	0.867	0.668	0.783	0.849	0.269
**Visual learning**	*r*	-0.062	0.303	0.249	-0.127	-0.153	0.335
	p	0.806	0.222	0.320	0.679	0.619	0.264
**Reasoning/problem-solving**	*r*	-0.061	-0.021	0.290	0.036	-0.343	0.238
	p	0.810	0.934	0.243	0.906	0.251	0.433
**Social cognition**	*r*	-0.361	0.120	0.278	0.149	-0.169	0.507
	p	0.142	0.636	0.264	0.627	0.580	0.077
**Overall composite score**	*r*	-0.135	0.103	0.166	-0.034	-0.040	0.346
	p	0.595	0.685	0.511	0.913	0.897	0.247

Abbreviations: ANBP, anorexia nervosa, binge-eating/purging type subtype; ANR, anorexia nervosa, restricting subtype; BMI, body mass index; EDE-Q, Eating Disorder Examination-Questionnaire; MCCB-J, MATRICS Consensus Cognitive Battery, Japanese-language version.

All correlation coefficients are Pearson coefficients. We defined statistical significance as $p < 0.05$.
