# Supplementary material for: Neurocognitive Impairments Are More Severe in the Binge-Eating/Purging Anorexia Nervosa Subtype Than in the Restricting Subtype
Source: Front Psychiatry. 2018 Apr 16;9:138. doi: 10.3389/fpsyt.2018.00138 (PMC5911723; doi:10.3389/fpsyt.2018.00138)
Supplement: Supplementary file 4 [file Image1.pdf]

Supplementary Figure 1. A, B : Partial correlation between chart-recorded minimum BMIs and MCCB-J overall composite scores for the ANR and ANBP groups. C, D : Partial correlation between BMIs at assessment and MCCB-J overall composite scores for the ANR and ANBP groups. E, F : Partial correlation between illness durations and MCCB-J overall composite scores for the ANR and ANBP groups. All partial correlation coefficients have been calculated with the three demographic variables (i.e., IQ, age, and years of education) as control variables.

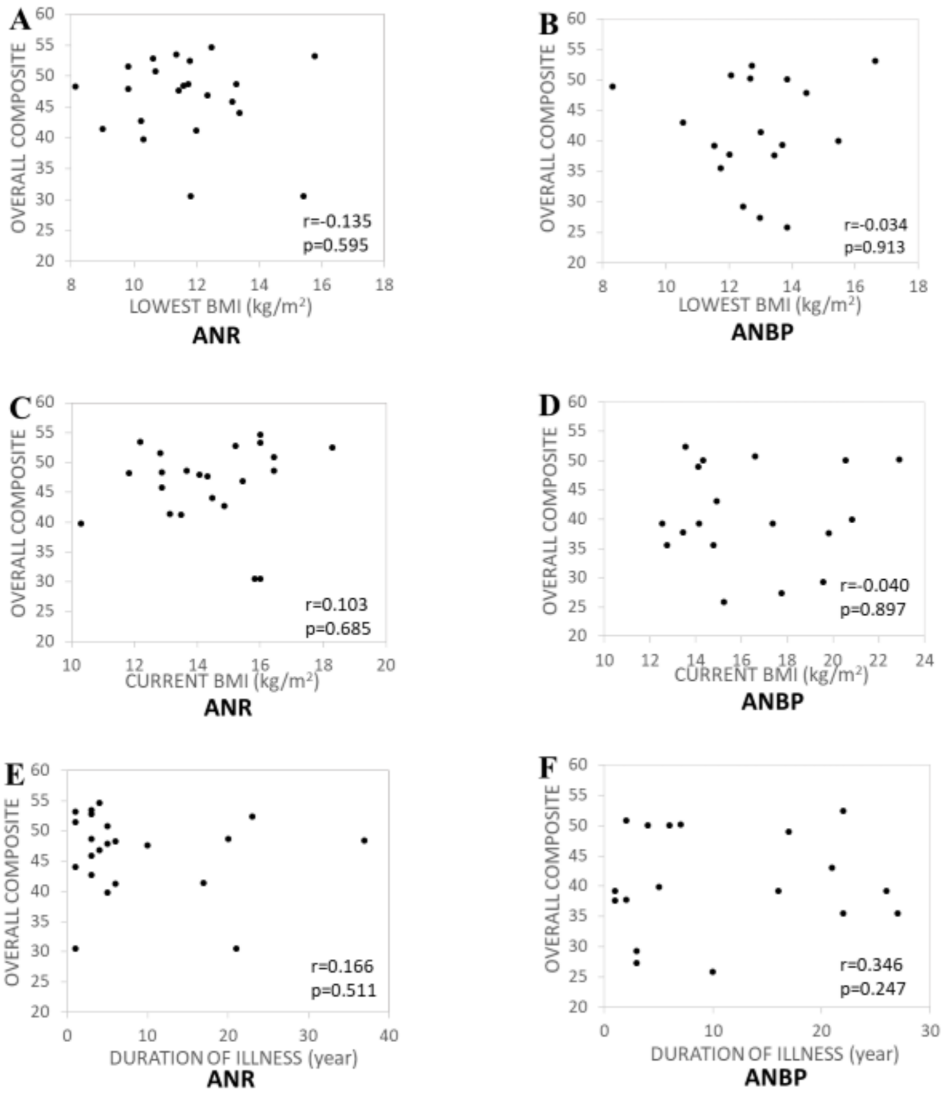

Abbreviations: ANBP, anorexia nervosa, binge-eating/purging subtype; ANR, anorexia nervosa, restricting subtype; BMI, body mass index; MCCB-J, MATRICS Consensus Cognitive Battery, Japanese-language version.
